# HeLa cells cross-contaminated nasopharyngeal carcinoma cell lines: Still a common problem

**DOI:** 10.1038/s41416-024-02675-x

**Published:** 2024-04-25

**Authors:** Anna Makowska, Udo Kontny, Ralf Weiskirchen

**Affiliations:** 1 Division of Pediatric Hematology, Oncology and Stem Cell Transplantation, University Hospital Aachen, Aachen, Germany; 2 Institute of Molecular Pathobiochemistry, Experimental Gene Therapy and Clinical Chemistry (IFMPEGKC), University Hospital Aachen, Aachen, Germany

**Keywords:** Cell biology, Diseases

## To the Editor:

We were intrigued by the article titled “*A Metabolic Map and Artificial Intelligence-Aided Identification of Nasopharyngeal Carcinoma via a Single-Cell Raman Platform*,” authored by Xu et al. [1]. In their study, the authors employed a single-cell Raman platform to create a metabolic map encompassing the following: (A) nasopharyngeal carcinoma (NPC) cell lines: They analyzed seven “authenticated” NPC cell lines and one immortalized nasopharyngeal epithelial cell line; (B) Tissue samples: The study included six nasopharyngeal mucosa tissues and seven NPC tissue samples. Through confocal Raman spectroscopic measurements and imaging, the researchers explored altered metabolic processes involving nucleic acids, amino acids, lipids, and sugars. Additionally, they investigated metabolomic profiles using ultra-performance liquid chromatography-tandem mass spectrometry (UPLC-MS/MS). Notably, the authors proposed that confocal Raman spectroscopy, along with imaging, serves as a highly sensitive diagnostic tool for classifying NPC cells and tissue. While the study provides a wealth of experimental data, it is essential to acknowledge that more than half of the cell lines used in this research have been misidentified as NPC cells, as reported in the Register of Misidentified Cell Lines (version 12, released on January 16, 2023) (Table 1). Out of the seven cell lines analyzed, five exhibit a high degree of genetic overlap with the HeLa short tandem repeat (STR) profile, specifically the CNE1, CNE2, SUNE1, 6-10B, and 5-8F lines. It is acknowledged that the CNE1, CNE2, and SUNE1 lines were initially derived from nasopharyngeal carcinoma. However, due to prolonged culturing and suboptimal laboratory practices, these lines have become contaminated with HeLa cells. Furthermore, the SUNE-1 line serves as the progenitor for the 6-10B and 5-8F lines, which, regrettably, also show genetic similarities to the HeLa STR profile.

**Table 1:** Summary of nasopharyngeal carcinoma cell lines used.

Cell line	ICLAC Registration ID	Cellosaurus	Comment^a^	References
SUNE-1	ICLAC-00595	CVCL_6946	This cell line has a high similarity to HeLa but a comparison of STR profiles gives an indeterminate result (<80% match). It appears to be either a hybrid cell line or a stable mixture between HeLa and another, unknown cell line. Although its identity has not been fully resolved, the cell line is either cross-contaminated or misidentified and should not be used as a model for nasopharyngeal carcinoma.	[3]
6-10B	ICLAC-00597	CVCL_C529	SUNE-1 derivative	[3]
5-8F	ICLAC-00596	CVCL_C528	SUNE-1 derivative	[3]
CNE-1	ICLAC-00473	CVCL_6888	STR profiling showed similarity to HeLa, but with less than an 80% match. Further data confirmed this, revealing that the cells contained HPV-18, as seen in HeLa cells. The authors suggest that a fusion may have taken place between HeLa and an unidentified cell. While not definitively proven, the current data align with this theory.	[2, 8]
CNE-2	ICLAC-00474	CVCL_6889	see CNE-1	[2, 8]
C666-1	NA	CVCL_7949	This subclone is derived from cell line C666, which originated from an NPC xenograft of Southern Chinese origin and contains a complete EBV genome.	[7]
HK1	NA	CVCL_7084	The NPC/HK1 cell line is derived from a well-differentiated squamous carcinoma with no demonstrable Epstein-Barr nuclear antigen (EBNA).	[9]

^a^data on SUNE-1, 6-10B, 5-8F, CNE-1, and CNE-2 were taken from the ICLAC database [10].

The issue of cross-contamination in nasopharyngeal carcinoma cell lines has been a recognized concern since 2008 and has been consistently emphasized in subsequent literature [2, 3]. The latest release from the International Cell Line Authentication Committee (ICLAC), their 12^th^ edition, identifies eight “NPC cell lines” as compromised by cross-contamination [4]. Despite clear genetic evidence indicating the presence of HeLa cell contamination, many research groups focusing on NPC have persisted in utilizing these compromised cell lines to date.

Improving cell line selection and reporting is crucial for research accuracy. However, the prevalence of publications with incorrect cell line data is concerning, as it leads to their continued use and risks the validity of research [5, 6]. Addressing this issue is essential for ensuring reliable scientific outcomes. It is important to establish stringent verification processes and foster a culture of transparency in research practices. Mandatory methods such as short tandem repeat (STR) profiling and DNA fingerprinting are needed to prevent cell line misidentification and safeguard scientific integrity.

The results presented show no significant differences between HeLa-contaminated cell lines and uncontaminated ones. Therefore, the method used does not appear to distinguish between tumor cell lines of various epithelial origins. However, in the study by Xu et al., the presence of HeLa contamination in NPC cell lines does not necessarily invalidate the research findings. Along with the contaminated lines, two uncontaminated NPC cell lines, HK1 (also known as NPC/HK1) and C666-1, were also used. The C666-1 line holds particular importance in NPC research as it consistently carries EBV, reflecting the viral patterns found in most primary NPC biopsies from Chinese patients [7]. The utilization of actual tissue samples further bolsters the study’s validity. Nevertheless, accurate identification of cell lines is crucial to prevent data misinterpretation.

## References

[1] Xu J, Chen D, Wu W, Ji X, Dou X, Gao X, et al. A metabolic map and artificial intelligence-aided identification of nasopharyngeal carcinoma via a single-cell Raman platform. Br J Cancer. 2024. 10.1038/s41416-024-02637-3 10.1038/s41416-024-02637-3 PMC11091122 38454165

[2] Chan SY, Choy KW, Tsao SW, Tao Q, Tang T, Chung GT (2008). Authentication of nasopharyngeal carcinoma tumor lines. Int J Cancer.

[3] Ye F, Chen C, Qin J, Liu J, Zheng C (2015). Genetic profiling reveals an alarming rate of cross-contamination among human cell lines used in China. FASEB J.

[4] American Type Culture Collection Standards Development Organization Workgroup ASN-0002. (2010). Cell line misidentification: the beginning of the end. Nat Rev Cancer.

[5] Weiskirchen S, Schröder SK, Buhl EM, Weiskirchen R (2023). A beginner’s guide to cell culture: Practical advice for preventing needless problems. Cells.

[6] Makowska A, Weiskirchen R (2024). Nasopharyngeal carcinoma cell lines: Reliable alternatives to primary nasopharyngeal cells?. Cells.

[7] Cheung ST, Huang DP, Hui AB, Lo KW, Ko CW, Tsang YS (1999). Nasopharyngeal carcinoma cell line (C666-1) consistently harbouring Epstein-Barr virus. Int J Cancer.

[8] Strong MJ, Baddoo M, Nanbo A, Xu M, Puetter A, Lin Z (2014). Comprehensive high-throughput RNA sequencing analysis reveals contamination of multiple nasopharyngeal carcinoma cell lines with HeLa cell genomes. J Virol.

[9] Huang DP, Ho JH, Poon YF, Chew EC, Saw D, Lui M (1980). Establishment of a cell line (NPC/HK1) from a differentiated squamous carcinoma of the nasopharynx. Int J Cancer.

[10] ICLAC Register of Misidentificed Cell Lines. Available at: https://iclac.org/ last accessed 16 March 2024.

